# Genetic Variants of the *TERT* Gene, Telomere Length, and Circulating *TERT* as Prognostic Markers in Rectal Cancer Patients

**DOI:** 10.3390/cancers12113115

**Published:** 2020-10-25

**Authors:** Enrica Rampazzo, Erika Cecchin, Paola Del Bianco, Chiara Menin, Gaya Spolverato, Silvia Giunco, Sara Lonardi, Sandro Malacrida, Antonino De Paoli, Giuseppe Toffoli, Salvatore Pucciarelli, Anita De Rossi

**Affiliations:** 1 Section of Oncology and Immunology, Department of Surgery, Oncology and Gastroenterology, University of Padova, Via Gattamelata 64, 35128 Padova, Italy; silvia.giunco@unipd.it (S.G.); anita.derossi@unipd.it (A.D.R.); 2 Experimental and Clinical Pharmacology Unit, Centro di Riferimento Oncologico (CRO)-IRCCS, Via Franco Gallini 2, 33081 Aviano (PN), Italy; ececchin@cro.it (E.C.); gtoffoli@cro.it (G.T.); 3 Clinical Research Unit, Veneto Institute of Oncology (IOV)-IRCCS, Via Gattamelata 64, 35128 Padova, Italy; paola.delbianco@iov.veneto.it; 4 Immunology and Molecular Oncology Unit, Veneto Institute of Oncology (IOV)-IRCCS, Via Gattamelata 64, 35128 Padova, Italy; chiara.menin@iov.veneto.it; 5 Section of Surgery, Department of Surgery, Oncology and Gastroenterology, Via Giustiniani 1, University of Padova, 35128 Padova, Italy; gaya.spolverato@unipd.it (G.S.); puc@unipd.it (S.P.); 6 Medical Oncology Unit 1, Veneto Institute of Oncology (IOV)-IRCCS, Via Gattamelata 64, 35128 Padova, Italy; sara.lonardi@iov.veneto.it; 7 Eurac Research, Institute of Mountain Emergency Medicine, Viale Druso Drususallee 1, 39100 Bolzano, Italy; sandro.malacrida@eurac.edu; 8 Radiation Oncology, Centro di Riferimento Oncologico (CRO)-IRCCS, Via Franco Gallini 2, 33081 Aviano (PN), Italy; adepaoli@cro.it

**Keywords:** TERT, telomerase, SNPs, variants, telomere length, circulating *TERT* mRNA, plasma, rectal cancer, neoadjuvant therapy, prognostic markers

## Abstract

**Simple Summary:**

Single nucleotide polymorphisms (SNPs) in the *TERT* gene, which encode the catalytic component of telomerase, can affect *TERT* expression and constitutive telomere length and have been associated with risk and/or outcome for several human cancers, but very few data are available about their impact on rectal cancer. The aim of our study was to comprehensively investigate the associations of eight common *TERT* SNPs with telomere length, circulating *TERT* mRNA in plasma, response to neoadjuvant therapy (CRT) and disease outcome in rectal cancer patients. Our findings indicate that the *TERT* variants can differently contribute to telomere erosion during CRT, circulating *TERT* levels and response to CRT. Thus, they could be a useful tool for improving the selection of patients who might benefit from CRT. Furthermore, circulating *TERT* variation during CRT and its level post-CRT are independent markers of response to CRT and disease progression.

**Abstract:**

Single-nucleotide polymorphisms (SNPs) in the *TERT* gene can affect telomere length and *TERT* expression and have been associated with risk and/or outcome for several tumors, but very few data are available about their impact on rectal cancer. Eight SNPs (rs2736108, rs2735940, rs2736098, rs2736100, rs35241335, rs11742908, rs2736122 and rs2853690), mapping in regulatory and coding regions of the *TERT* gene, were studied in 194 rectal cancer patients to evaluate their association with constitutive telomere length, circulating *TERT* mRNA levels, response to neoadjuvant chemoradiotherapy (CRT) and disease outcome. At diagnosis, the rs2736100CC genotype was associated with longer telomeres measured pre-CRT, while the rs2736100CC, rs2736108TT and rs2735940AA were associated with greater telomere erosion evaluated post-CRT. The rs2736108CC and rs2853690AA/GG genotypes, respectively associated with lower telomere erosion and lower levels of circulating *TERT* post-CRT, were also independently associated with a better response to therapy [OR 4.6(1.1–19.1) and 3.0(1.3–6.9)]. Overall, post-CRT, low levels (≤ median value) of circulating *TERT* and its stable/decreasing levels compared to those pre-CRT, were independently associated with a better response to therapy [OR 5.8(1.9–17.8) and 5.3(1.4–19.4), respectively]. Furthermore, post-CRT, patients with long telomeres (>median value) and low levels of circulating *TERT* had a significantly lower risk of disease progression [HR 0.4(0.1–0.9) and 0.3(0.1–0.8), respectively]. These findings suggest that *TERT* SNPs could be a useful tool for improving the selection of patients who could benefit from CRT and support the role of telomere length and circulating *TERT* mRNA levels as useful markers for monitoring the response to therapy and disease outcome in rectal cancer patients.

## 1. Introduction

Telomeres, composed of the TTAGGG repeat sequence, are special chromatin structures located at the end of each chromosome that maintain chromosomal integrity by protecting chromosome ends from DNA damage and end-to-end fusions [1]. Human telomeres lose between 50–200 base pairs with each replication, and when telomere erosion reaches a critical point, cells cease to proliferate and undergo senescence or apoptosis [2]. To overcome this proliferation barrier, tumor cells must stabilize their telomeres. Telomere length is maintained by telomerase, a ribonucleoprotein complex containing an internal RNA component and a catalytic protein, Telomerase Reverse Transcriptase (TERT), with telomere-specific reverse transcriptase activity [3]. TERT, which synthesizes *de novo* telomere sequences using internal RNA as a template, is the rate-limiting component of the telomerase complex, and its expression is correlated with telomerase activity [4].

The *TERT* gene is located on chromosome 5p15.33 and is strictly regulated by the transcriptional activity of the promoter region [5]. Most normal somatic cells do not display telomerase activity, whereas a high level of telomerase activity is detected in germinal cells, immortalized cell lines, and 85–90% of human cancers [6]. Recent studies have identified several single nucleotide polymorphisms (SNPs) in the *TERT* gene, including regulatory regions, which can affect *TERT* expression [7,8,9,10] and constitutive telomere length [11,12,13] and have been associated with risk and/or outcome for several human cancers [14,15,16,17]. Nevertheless, to date, previous studies on colorectal cancer have evaluated the association of genetic *TERT* variants with cancer risk [18,19,20], but not with clinical outcome.

Rectal cancer accounts for approximately one third of all colorectal cancers and is associated with worse clinical outcome [21]. The standard treatment of locally advanced rectal cancer is pre-operative neoadjuvant chemoradiotherapy (CRT) followed by a radical surgery. The degree of response to CRT varies among patients and pathological complete response is associated with better outcome [22,23]. To date, there is an essential need for biomarkers that can predict response to CRT at an early time point, allowing the selection of rectal cancer patients who would or would not benefit from CRT, to provide adequate treatment option and to reduce toxicity associated with ineffective CRT. Recently, it has been demonstrated that plasma *TERT* mRNA levels may serve as a minimally invasive marker for monitoring response to therapy in rectal cancer patients who underwent pre-operative chemoradiotherapy [24,25]; nevertheless, to our knowledge, the role of genetic *TERT* variants in constitutive telomere length and in *TERT* mRNA expression, and their associations with response to neoadjuvant therapy and outcome in rectal cancer patients, have not yet been investigated. In the present study, we studied patients with rectal cancer to comprehensively investigate the associations of eight common *TERT* SNPs with telomere length, circulating *TERT* mRNA in plasma, response to CRT and disease outcome. This information could constitute the basis for useful tools in clinical practice.

## 2. Results

### 2.1. Clinical and Demographic Characteristics of Rectal Cancer Patients

This study was conducted in 194 patients with primary locally advanced rectal carcinoma, who were enrolled in a prospective study and underwent preoperative CRT, followed by either total mesorectal or local excision. The characteristics of the 194 patients are summarized in Table 1.

### 2.2. Association between SNP Genotypes and Telomere Length in Peripheral Blood Cells of Rectal Cancer Patients

**Figure 1:**
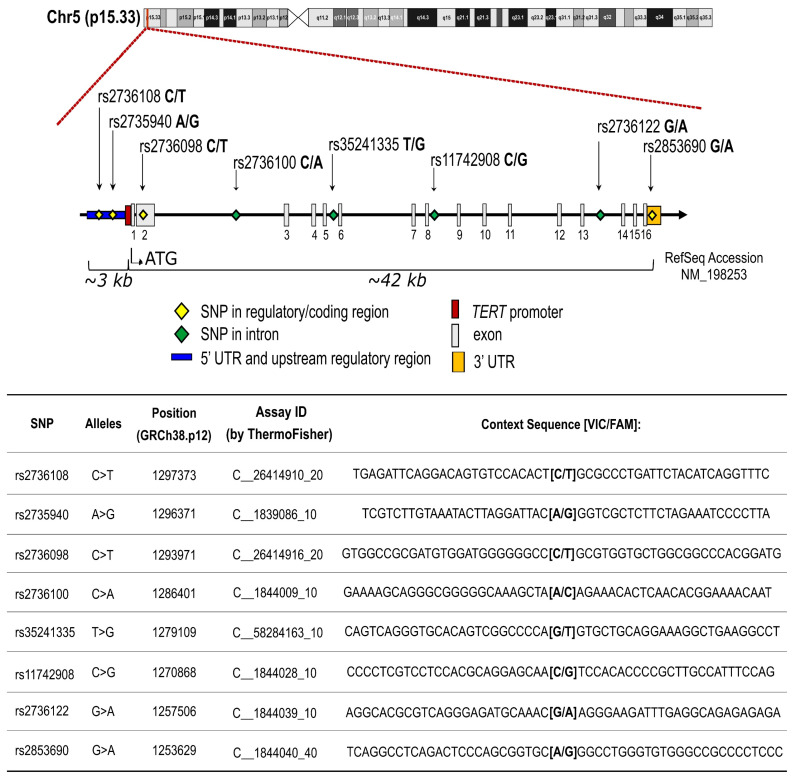
Schematic illustration and characteristics of selected SNPs within *TERT* gene.

Eight SNPs in *TERT* gene (rs2736108, rs2735940, rs2736098, rs2736100, rs35241335, rs11742908, rs2736122, and rs2853690) were genotyped using DNA from peripheral blood cells from all patients (see the Materials and Methods section for details). Gene position and characteristics of selected SNPs are shown in Figure 1.

Telomere length was measured in all cases at diagnosis, pre-CRT (T0), and was significantly inversely correlated with age (r_s_ = −0.162; *p* = 0.024) (Figure S1, Supplementary Materials). The telomere lengths did not significantly differ with tumor stage or grading.

**Figure 2:**
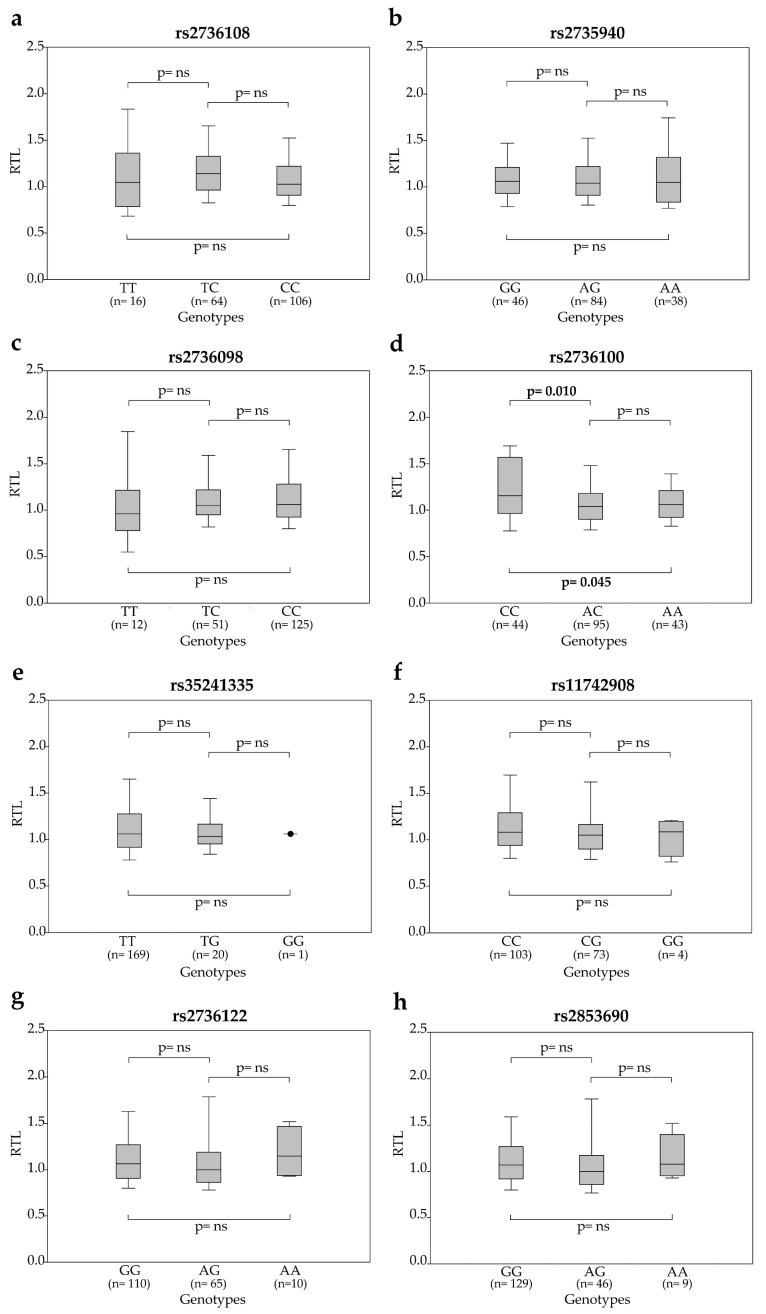
Relative telomere length (RTL) at T0 according to SNP genotypes: (**a**) rs2736108, (**b**) rs2735940, (**c**) rs2736098, (**d**) rs2736100, (**e**) rs35241335, (**f**) rs11742908, (**g**) rs2736122 and (**h**) rs2853690. Boxes and whiskers: 25–75th and 10–90th percentiles, respectively; central line in boxes: median.

The rs2736100CC genotype was significantly associated with longer telomeres [1.15 (0.97–1.54)] compared to AC [1.04 (0.90–1.17); *p* = 0.010] and AA [1.06 (0.93–1.18); *p* = 0.045] genotypes (Figure 2). Post-CRT (T2), at the time of surgery, peripheral blood cells were available for a subgroup of 74 patients. The distribution of RTL according to SNP genotypes is shown in Table S1A (Supplementary Materials). The ∆RTL (RTL at T2 minus RTL at T0) distribution within SNP genotypes showed significant associations between telomere shortening and rs2736100CC [−0.15 (−0.49–0.10)], rs2735940AA [−0.33 (−0.68–0.26)] and rs2736108TT [−0.35 (−0.65–0.20)] genotypes (Table S1B, Supplementary Materials), thus suggesting a potential link between these genotypes and the erosion of telomeres under CRT.

### 2.3. Association between SNP Genotypes and Circulating TERT mRNA Levels

**Figure 3:**
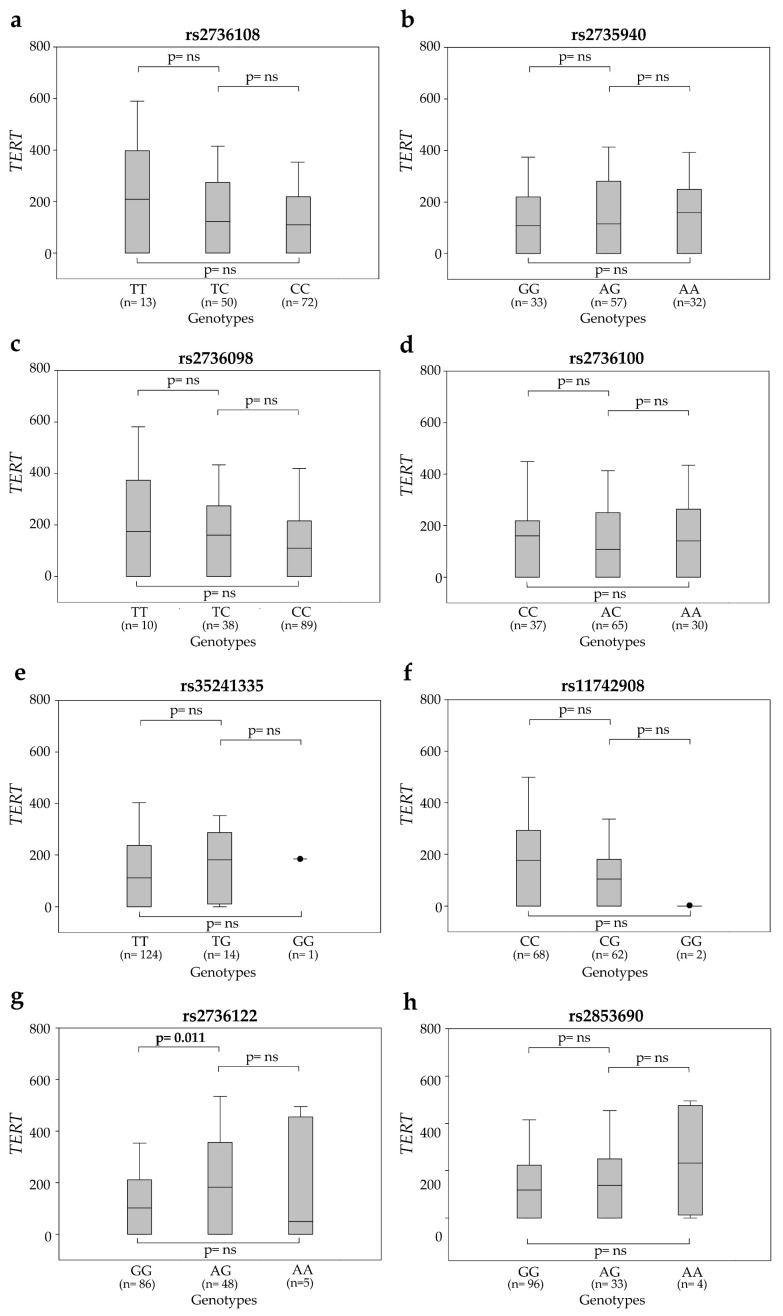
Levels of circulating *TERT* mRNA at T0 according to SNP genotypes: (**a**) rs2736108, (**b**) rs2735940, (**c**) rs2736098, (**d**) rs2736100, (**e**) rs35241335, (**f**) rs11742908, (**g**) rs2736122 and (**h**) rs2853690. Boxes and whiskers: 25–75th and 10–90th percentiles, respectively; central line in boxes: median.

The distribution of circulating *TERT* mRNA levels at T0 according to SNP genotypes is shown in Figure 3. At T0, the rs2736122AG carriers showed higher circulating *TERT* mRNA levels compared to patients with rs2736122GG genotype [182 (0.0–352) vs. 102 (0.0–208.5); *p* = 0.011]. The levels of *TERT* mRNA did not significantly differ with tumor stage or grading. At T2, plasma samples were available for 122 patients. Overall, circulating *TERT* levels were lower than those observed at T0 (median 45 vs. 122 copies/mL). In particular, rs2853690AG was associated with significantly higher levels of circulating *TERT* than the other genotypes (131 vs. 25 and vs. 0 copies/mL, *p* = 0.016 and *p* = 0.108, respectively) (Table S2A, Supplementary Materials). However, the distribution of ∆*TERT* (*TERT* levels at T2 minus *TERT* levels at T0) did not significantly differ among the SNP genotypes (Table S2B, Supplementary Materials).

### 2.4. Association of SNP Genotypes, Telomere Length and Circulating Plasma TERT mRNA with Response to Neoadjuvant Therapy

**Figure 4:**
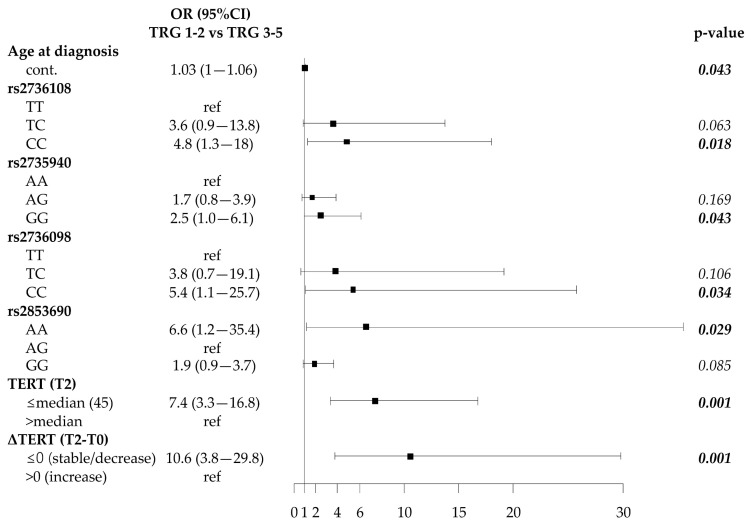
Forest plot showing odds ratios (OR) according to tumor regression grading (TRG) probability using univariate logistic regression analysis. Cont.: continuous variable.

Patients were divided according to the simplified TRG classification: 90 patients (56 TRG1, 34 TRG2) were responders, and 104 patients (83 TRG3, 18 TRG4, 3 TRG5) were non-responders (Table S3A, Supplementary Materials). The responders were significantly older than non-responders [66 (61–72) vs. 63 (57–71); *p* = 0.043], and no other significant differences were found in the other baseline characteristics (Figure 4 and Table S3A, Supplementary Materials). The frequency distribution of *TERT* SNPs showed that the rs2736108CC, rs2735940GG, rs2736098CC genotypes were more prevalent in responder than in non-responder patients (63.6% vs. 51.0%; 32.9% vs. 22.5%; 73.0% vs. 60.6%, respectively). In the codominant model these genotypes were significantly associated with a better response to CRT (rs2736108: CC vs. TT, *p* = 0.018; rs2735940: GG vs. AA, *p* = 0.043; rs2736098: CC vs. TT, *p* = 0.034) (Figure 4 and Table S3B, Supplementary Materials). In addition, both the GG and AA genotypes of the rs2853690 tended to be more associated with a better response to CRT compared to the AG genotype (Figure 4 and Table S3B, Supplementary Materials). Haplotype analysis, including all SNPs, showed that haplotype rs2736108C-rs2735940G-rs2736098C-rs2736100A-rs35241335T-rs11742908C-rs2736122A-rs2853690G was associated with the best response to CRT (Table S4A, Supplementary Materials). The haplotype analysis involving the haploblock of the 3 SNPs rs2736108, rs2735940 and rs2736098 showed that the corresponding T-A-T haplotype was associated with a worse response to CRT compared to the reference C-G-C haplotype (OR = 0.45; 95%CI 0.26–0.80; *p* = 0.007) (Table S4B, Supplementary Materials), thus supporting the suggestion of the “protective” role of C-G-C haplotype. Overall, telomere length and circulating *TERT* levels measured pre-CRT (T0) were not associated with response to CRT. At T2, responder patients had longer telomeres (RTL > median value) than non-responders (56.2% vs. 42.9%), but not at a significant level (Table S3B, Supplementary Materials). Notably, at T2, more responders had lower circulating *TERT* levels (≤median value) than non-responder patients (76.9% vs. 31.0%, *p* < 0.001); moreover, 90.2% of responders had stable/decreased *TERT* levels (Δ*TERT*≤ 0) compared to 46.5% of non-responder patients (*p* < 0.001) (Figure 4 and Table S3B, Supplementary Materials).

The multivariable analysis, using the significant variables at T0 in the univariate analysis, confirmed the prognostic role of rs2736108CC and rs2853690AA/GG genotypes (*p* = 0.034 and *p* = 0.008; respectively) at diagnosis, pre-CRT (Table 2). Notably, rs2736108CC was associated with less erosion of telomeres (Table S1B, Supplementary Materials). The rs2853690 is of particular interest: the AG genotype, less “protective” than GG and AA genotypes, is associated with higher levels of circulating *TERT* (Table S2A, Supplementary Materials). The multivariable analysis, using the significant variables at T2, showed that low *TERT* levels (< median value) and stable/decreased *TERT* levels (Δ*TERT* ≤ 0) were associated with a better response to CRT (*p* = 0.002 and *p* = 0.012, respectively) (Table 2).

### 2.5. Associations of SNP Genotypes, Telomere Length and Circulating TERT mRNA with Disease Outcome

**Figure 5:**
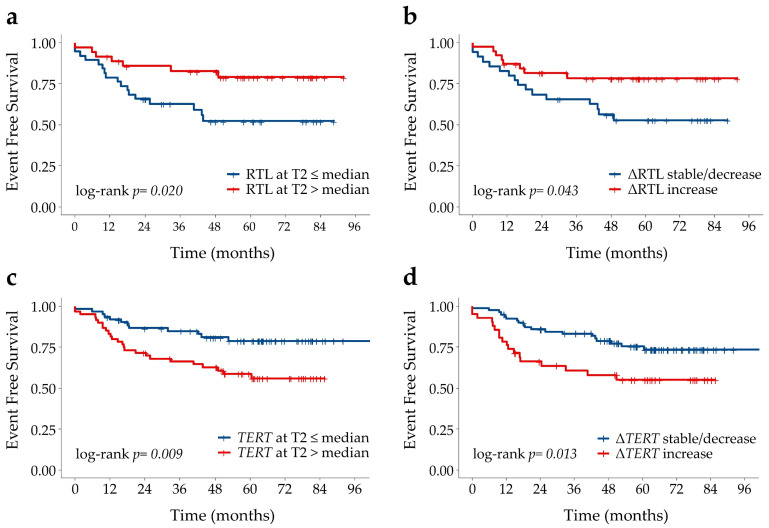
Kaplan-Meier curves for event-free-survival according to (**a**) relative telomere length (RTL) above or below the median at T2, (**b**) ∆RTL (T2-T0) stable/decrease or increase zero, (**c**) *TERT* levels above or below the median at T2 and (**d**) ∆*TERT* mRNA (T2-T0) stable/decrease or increase.

Overall, none of the SNP genotypes were associated with disease outcome (Table S5, Supplementary Materials). Notably, while telomere length at baseline was not associated with outcome, telomere length at T2 correlated with the event free survival (EFS). Indeed, post-CRT patients with long telomeres (RTL > median value), and no telomere erosion during CRT (∆RTL > 0), had a lower risk of a negative event than patients with short telomeres and ∆RTL ≤ 0 (*p* = 0.026 and *p* = 0.049, respectively, Table S5, Supplementary Materials), thus suggesting that telomere erosion during CRT could contribute to increasing the risk of a negative event. Patients with low circulating *TERT* levels (≤ the median) at T2 had almost 60% lower risk of a negative event than patients with high *TERT* levels (> the median) (*p* = 0.012) (Table S5, Supplementary Materials); moreover, patients who showed no increase in *TERT* levels during CRT (Δ*TERT* ≤ 0) had an almost 55% lower risk of a negative event than patients who underwent an increase in *TERT* levels (*p* = 0.016) (Table S5, Supplementary Materials). The patients’ EFS curves according to RTL and circulating *TERT* levels are shown in Figure 5. Multivariable analysis confirmed that high RTL and low circulating *TERT* levels after CRT were independent prognostic factors of a better outcome: patients with longer telomere and lower *TERT* levels at T2 had a lower risk of developing negative events (*p* = 0.024 and *p* = 0.015; respectively, Table 3).

## 3. Discussion

SNPs in the *TERT* gene have been associated with outcomes in several cancers [15,16,17]. However, to date no data have been available on the association between *TERT* genetic variants and response to neoadjuvant therapy and outcome in rectal cancer patients. To our knowledge, our study is the first to address these issues.

First of all, in agreement with other studies [26], we found that the rs2736100CC genotype is associated with a longer telomere length. Although the molecular mechanisms by which the rs2736100C allele influences telomere length are presently not well understood, it was found to be associated with increased *TERT* transcription [8,27]. Moreover, a previous study suggested that this SNP is located in a regulatory region of the *TERT* gene in linkage disequilibrium with other nearby variants which could affect *TERT* expression, and thus telomere length [28]. Interestingly, in our study, patients with rs2736100CC genotype had longer telomere at diagnosis and greater telomere erosion under CRT compared to the patients with the other genotypes. This finding may suggest that a longer telomere could be more exposed to the effects of radiation and drugs employed during CRT. Several studies have shown that chemotherapeutic drugs induce the shortening of constitutive telomeres in peripheral blood cells [29,30]. It has been shown that the toxicity induced by 5-fluorouracil (5-FU) on hematopoietic cells was associated with a shortening of telomere length in peripheral blood mononuclear cells [31]. 5-FU exerts its anticancer effects through inhibition of thymidylate synthase and interferes with the synthesis of DNA and RNA [32]. Therefore, it is conceivable that it could affect telomere length, regardless of *TERT* expression.

In our cohort, the patients carrying the rs2736108TT and rs2735940AA genotypes, two SNPs mapping in the promoter, also showed a greater telomere shortening under CRT compared to the patients with the other genotypes. It has been suggested that chemotherapy-induced telomere shortening is related to cellular replication rate [33], thus it is conceivable that mononuclear cells of patients with these SNP genotypes had a higher replication rate, and therefore a higher telomere erosion under CRT. Telomere shortening can lead to chromosomal instability, which in turn leads to a poor response to CRT and to tumor progression. The finding that the rs2736108CC and rs2735940GG genotypes favor a better response to therapy is consistent with the observation that the rs2736108TT and rs2735940AA variants underwent greater telomere erosion under CRT. Our finding that lower telomere erosion during CRT and longer telomeres post-CRT are associated with a lower risk of a negative event are consistent with the meta-analysis on colorectal cancer indicating that short telomeres in peripheral blood cells are independently associated with poorer overall survival [34].

An important and new result of this study is that at diagnosis, the SNP genotypes rs2736108CC, rs2735940GG, rs2736098CC, and rs2853690AA/GG were associated with a better response to neoadjuvant therapy, and their protective role was supported by the evidence that both full length C-G-C-A-T-C-A-G and the short C-G-C haplotypes were more frequent in CRT responders than in non-responders. The “protective” effect of SNPs rs2736108CC and rs2853690AA/GG was confirmed by the multivariable analysis. The downregulation of *TERT* transcription can significantly contribute to the efficacy of chemotherapy treatment [35]. In this context, it is of interest to note that rs2853690AG, associated with a worse response to CRT, was also associated with higher levels of circulating *TERT* mRNA compared to the AA and GG genotypes.

As mentioned before, the role of SNPs is probably dependent on their involvement in *TERT* expression [7,8,9,10]. However, in our study we found that at baseline only the SNP rs2736122AG was significantly associated with a higher level of circulating *TERT* compared to the other genotypes, while at T2 only the rs2853690AA/GG “protective genotypes” were associated with low levels of circulating *TERT*. It should be stressed that in this study we analyzed the relationship between SNPs and circulating *TERT* for the first time. The lack of a strong significant association between *TERT* polymorphisms and circulating *TERT* mRNA levels may be explained by the fact that plasma *TERT* mRNA derives from apoptosis/necrosis of tumor cells, where several other molecular pathways can affect telomerase expression. Nonetheless, as demonstrated previously [24], we found that circulating *TERT* levels at T2 and Δ*TERT* levels were predictive of tumor response and prognostic of disease outcome. This is not surprising in light of accumulating evidence suggesting that, besides its canonical role in stabilizing telomeres, *TERT* may promote tumorigenesis through extra-telomeric functions, including enhancement of proliferation, resistance to apoptosis, inflammation, invasion and metastasis altogether contributing towards the higher resistance of cancer cells [36,37,38]. Therefore, endowed with these non-telomeric functions, *TERT* can participate in all the major characteristics of the cancer phenotype, thereby supporting its role as a potential prognostic tumor marker.

## 4. Materials and Methods

### 4.1. Samples

The 194 rectal cancer patients enrolled in this study underwent CRT and surgery at the Surgery Section, Department of Surgery, Oncology and Gastroenterology, University of Padova (133 patients), and at the Centro di Riferimento Oncologico, Aviano (61 patients). Peripheral blood samples were available from all 194 patients at diagnosis, pre-CRT (T0), and for 74 patients at (−2 to 0 day) surgery occurring after CRT (T2). Data on circulating *TERT* mRNA levels in plasma were partially available from our previous study [24]. The local Ethics Committees approved the study (protocol N. 35333/AO/15), and each patient signed the informed consent.

### 4.2. Evaluation of Pathologic Tumor Response

The evaluation of pathologic tumor response was assessed as previously described [24,25]. Briefly, the tumors were pathologically staged according to the American Joint Committee on Cancer that established the TNM staging system [39,40]. The tumor response to CRT was assessed in the surgical specimens and categorized according to Mandard’s tumor regression grading (TRG) system [41]. Patients with TRG1 and TRG2 were classified as responders, and those with TRG 3 to 5 as non-responders.

### 4.3. DNA Extraction from Peripheral Blood Cells

Peripheral blood samples from patients enrolled at the University of Padova were centrifuged at 3000× *g* for 10 min at room temperature in a Megafuge 1.0 R (Hareaus, Hanau, Germany) and buffy coats were stored at −80 °C until use. DNA was extracted using QIAmp DNA Blood Mini Kit (Qiagen, Hilden, Germany) according to the manufacturer’s instructions. Whole blood samples collected from patients enrolled at CRO Aviano were used for genomic DNA extraction on an automated EZ1 DNA Extractor (Qiagen).

### 4.4. Telomere Length and TERT mRNA Quantification

Relative telomere length (RTL) was determined by multiplex quantitative real-time PCR, as previously described [42,43]. Each PCR reaction was performed in a final volume of 25 μL, containing 5 μL sample (10 ng DNA) and 20 μL master mix ready-to-use 1× Light Cycler 480 SYBR Green I (Roche Life Science, Penzberg, BY, Germany), containing 900 nmol/L of each primer. Sequences of telomere and albumin primers and the thermal cycling profile are detailed in previous studies [42,43]. A standard curve was generated at each PCR run, consisting of DNA from the RAJI cell line, serially diluted from 10 to 0.41 ng/µL [44]. All DNA samples and reference samples were run in triplicate. The LC480Conversion and the LinRegPCR free software were used to convert raw text files and to analyze the converted data. RTL values were calculated as a telomere/single-copy gene ratio, as previously described [44]. *TERT* mRNA in plasma was quantified exactly as previously detailed [24].

### 4.5. SNP Selection and Genotyping

A haplotypic block analysis approach was used applied on the *TERT* gene, including 3000 bases in upstream and downstream areas. A list of the SNPs tagging each haploblock was downloaded from the SNPinfo website using a specific tool (https://snpinfo.niehs.nih.gov/snpinfo/snptag.html). The criteria for the selection were: (1). a minor allelic frequency of at least 5%; (2). the tagged haploblock includes at least two SNPs; (3). an available real-time TaqMan assay. The SNPs selected were the following: rs2736098, rs35241335, rs11742908, rs2736122 and rs2853690. Three additional SNPs were included based on relevant literature data regarding their potential functional effect of the SNP: rs2736108 and rs2735940 mapping in the promoter region, and rs2736100 in the intron 2 (Figure 1). All eight SNPs were genotyped by an allelic discrimination method using predesigned TaqMan SNP genotyping assays. All commercial TaqMan assays were purchased from Applied Biosystems (Foster City, CA, USA) by ThermoFisher Scientific (https://www.appliedbiosystems.com) and the analyses were performed using the Applera TaqMan Universal Master mix on an Applied Biosystems ABI 7500 instrument, according to the manufacturer’s instructions. For each genotype, control samples, confirmed by Sanger-sequencing, were included in each analysis.

### 4.6. Statistical Analyses

For genotype analyses, major and minor allele frequencies in the European ancestry population were considered according to the dbSNP database (https://www.ncbi.nlm.nih.gov/snp/rs6065). Patients’ characteristics were reported as median and interquartile range (IQR) for quantitative data, and frequencies and percentages for categorical data. The probability of response to neoadjuvant therapy was explored using a logistic regression model. The results are shown as odds ratios (OR) with 95% confidence interval (CI). Multivariable logistic regression analysis was used to determine the adjusted association of factors on the probability of response. Haplotype analysis was performed with the SNPStats program (https://www.snpstats.net/start.htm), which offers the possibility of estimating haplotype frequencies from genotype frequencies using the Expectation-Maximization (EM) algorithm coded into the haplo.stats package [45]. The association analysis of the haplotypes to response to neoadjuvant therapy was similarly performed for genotype analysis. Logistic regression results are shown as OR with 95% CI. The most frequent haplotype was automatically selected as the reference category and rare haplotypes were pooled together in a group. EFS was evaluated for all patients with the time from sample collection to a negative event being defined as relapse, progression, or death. Patients who did not develop any event during the study period were censored at the date of last observation. The EFS probabilities were estimated using the Kaplan-Meier method and compared among strata using the log-rank test. Telomere length and *TERT* mRNA were dichotomized with their median value, while changes were considered as increases if higher or stable/decrease. The hazard ratios (HR) with 95% CI were obtained from univariate and multiple Cox proportional hazards regression models. No deviation from the proportional hazards assumption was found using the test statistic of Grambsch and Therneau [46]. All tests were two-sided and a *p*-value < 0.05 was considered to be statistically significant. Statistical analyses were performed using SAS version 9.4 (SAS Institute, Cary, NC, USA) and RStudio (RStudio: Integrated Development for R. RStudio, Inc., Boston, MA, USA).

## 5. Conclusions

In conclusion, our findings indicate for the first time that SNPs of the *TERT* gene may be a useful tool in diagnosis to improve the selection of patients who could benefit from CRT. Furthermore, they play different roles in telomere erosion during CRT, thus suggesting a potential impact on telomere shortening, a risk factor of tumor progression. Finally, the *TERT* variation during CRT and its level post-CRT are important independent markers of response to CRT and disease progression.

## Figures and Tables

**Table 1:** Clinical and demographic characteristics of rectal cancer patients.

Characteristics		Total
		(*n* = 194)
Age at diagnosis (years)	Median (IQR)	65 (58–72)
Gender	M	138 (71.1%)
	F	56 (28.9%)
Distance from anal verge(cm)	Median (IQR)	5 (3–8)
	missing	9
CEA, ng/mL (T0)	Median (IQR)	2.0 (1.2–3.5)
	Missing	45
Grading	G1	12 (7.7%)
	G2	132 (84.6%)
	G3	12 (7.7%)
	Missing	38
cTNM	I	6 (3.2%)
	II	21 (11.1%)
	III	159 (84.1%)
	IV	3 (1.6%)
	Missing	5
Total RT dose	<50.4 Gy	22 (12.4%)
	50.4 Gy	89 (50.0%)
	>50.4 Gy	67 (37.6%)
	Missing	16
Fluoropyrimide	Alone	93 (50.8%)
	+other drugs	84 (45.9%)
	No	6 (3.3%)
	Missing	11
Interval between CRT and surgery (months)	Median (IQR)	1.9 (1.7–2.2)
Interval between diagnosis and Surgery (months)	Median (IQR)	3.5 (3.1–3.9)

IQR = interquartile range; CEA = carcinoembryonic antigen; cTNM= clinical Tumor, Node, Metastasis classification; RT = radiotherapy; CRT = neoadjuvant chemoradiotherapy.

**Table 2:** Multivariable logistic regression analysis of TRG probability in rectal cancer patients pre and post-CRT.

**Variable Pre-CRT**	**Genotype**	**OR** **TRG 1-2 vs TRG 3-5** **(95% CI)**	***p*-Value**
Age at diagnosis (years)	cont.	1.03 (1.0─1.07)	*0.052*
rs2735940	AA		*ns*
	AG		
	GG		
rs2736098	TT		*ns*
	TC		
	CC		
rs2736108	TT	*Ref*	
	TC	3.4 (0.8─14.4)	*0.102*
	CC	4.6 (1.1─19.1)	***0.034***
rs2853690	GG	2.7 (1.2-6.3)	***0.017***
	AG	*Ref*	
	AA	9.7 (1.6─57.0)	***0.012***
	AA/GG	3.0 (1.3─6.9)	***0.008***
**Variable Post-CRT**	**Genotype/Characteristics**	**OR** **TRG 1-2 vs TRG 3-5** **(95% CI)**	***p*-Value**
Age at diagnosis (years)	cont.		*ns*
rs2735940	AA		*ns*
	AG		
	GG		
rs2736098	TT		*ns*
	TC		
	CC		
rs2736108	TT		*ns*
	TC		
	CC		
rs2853690	GG		*ns*
	AG		
	AA		
*TERT* (T2)	≤median (45)	5.8 (1.9─17.8)	***0.002***
	>median	*Ref*	
Δ*TERT* (T2-T0)	≤0 (stable/decrease)	5.3 (1.4─19.4)	***0.012***
	˃0 (increase)	*Ref*	

TRG = tumor regression grade; OR = odds ratio; CI = confidence interval; cont. = continuous variable.

**Table 3:** Multivariable Cox regression analysis indicating associations between covariates of interest and event free survival.

Covariate	Characteristics	HR (95%CI)	*p*-Value
RTL (T2)	≤median (1.14)	*Ref*	
	>median	0.4 (0.1–0.9)	***0.024***
ΔRTL (T2-T0)	≤0 (stable/decrease)		*ns*
	>0 (increase)		
*TERT* (T2)	≤median (45)	0.3 (0.1–0.8)	***0.015***
	>median	*Ref*	
Δ*TERT* (T2-T0)	≤0 (stable/decrease)		*ns*
	>0 (increase)		

HR = hazard ratio; CI = Confidence Interval; RTL = relative telomere length.

## Supplementary Materials

The following are available online at https://www.mdpi.com/2072-6694/12/11/3115/s1, Table S1A: Association between SNP genotypes and RTL in peripheral blood cells after neoadjuvant therapy, Table S1B: Association between SNP genotypes and RTL changes after neoadjuvant therapy, Table S2A: Association between SNP genotypes and *TERT* levels after neoadjuvant therapy, Table S2B: Association between SNP genotypes and *TERT* changes after neoadjuvant therapy, Table S3A: Univariate logistic regression analysis of clinical and demographical characteristics according to TRG probability in rectal cancer patients, Table S3B: Univariate logistic regression analysis of covariates of interest according to TRG probability in rectal cancer patients, Table S4A: Haplotype distribution in responder (TRG 1-2) and non-responders (TRG 3-5) to CRT, Table S4B: 5′ *TERT* gene haplotype distribution in responders (TRG 1-2) and non-responders (TRG 3-5) to CRT, Table S5: Univariate Cox regression analysis of EFS, Figure S1: Correlation between age and telomere length in peripheral blood cells of rectal cancer patients.

## References

[1] Blackburn E.H. (1991). Structure and function of telomeres. Nature.

[2] De Lange T. (2002). Protection of mammalian telomeres. Oncogene.

[3] Morin G.B. (1989). The human telomere terminal transferase enzyme is a ribonucleoprotein that synthesizes TTAGGG repeats. Cell.

[4] Nakamura T.M., Morin G.B., Chapman K.B., Weinrich S.L., Andrews W.H., Lingner J., Harley C.B., Cech T.R. (1997). Telomerase catalytic subunit homologs from fission yeast and human. Science.

[5] Cong Y.S., Wen J., Bacchetti S. (1999). The human telomerase catalytic subunit hTERT: Organization of the gene and characterization of the promoter. Hum. Mol. Genet..

[6] Shay J.W., Wright W.E. (2005). Senescence and immortalization: Role of telomeres and telomerase. Carcinogenesis.

[7] De Martino M., Taus C., Lucca I., Hofbauer S.L., Haitel A., Shariat S.F., Klatte T. (2016). Association of human telomerase reverse transcriptase gene polymorphisms, serum levels, and telomere length with renal cell carcinoma risk and pathology. Mol. Carcinog..

[8] Wei R., Cao L., Pu H., Wang H., Zheng Y., Niu X., Weng X., Zhang H., Favus M., Zhang L. (2015). TERT Polymorphism rs2736100-C Is Associated with EGFR Mutation-Positive Non-Small Cell Lung Cancer. Clin. Cancer Res..

[9] Sheng X., Tong N., Tao G., Luo D., Wang M., Fang Y., Li J., Xu M., Zhang Z., Wu D. (2013). TERT polymorphisms modify the risk of acute lymphoblastic leukemia in Chinese children. Carcinogenesis.

[10] Beesley J., Pickett H.A., Johnatty S.E., Dunning A.M., Chen X., Li J., Lu Y., Rider D.N., Palmieri R.T., Stutz M.D. (2011). Functional polymorphisms in the TERT promoter are associated with risk of serous epithelial ovarian and breast cancers. PLoS ONE.

[11] Codd V., Nelson C.P., Albrecht E., Mangino M., Deelen J., Buxton J.L., Hottenga J.J., Fischer K., Esko T., Surakka I. (2013). Identification of seven loci affecting mean telomere length and their association with disease. Nat. Genet..

[12] Mirabello L., Yu K., Kraft P., De Vivo I., Hunter D.J., Prescott J., Wong J.Y., Chatterjee N., Hayes R.B., Savage S.A. (2010). The association of telomere length and genetic variation in telomere biology genes. Hum. Mutat..

[13] Matsubara Y., Murata M., Yoshida T., Watanabe K., Saito I., Miyaki K., Omae K., Ikeda Y. (2006). Telomere length of normal leukocytes is affected by a functional polymorphism of hTERT. Biochem. Biophys. Res. Commun..

[14] Mocellin S., Verdi D., Pooley K.A., Landi M.T., Egan K.M., Baird D.M., Prescott J., De Vivo I., Nitti D. (2012). Telomerase reverse transcriptase locus polymorphisms and cancer risk: A field synopsis and meta-analysis. J. Natl. Cancer Inst..

[15] Ma R., Liu C., Lu M., Yuan X., Cheng G., Kong F., Lu J., Strååt K., Björkholm M., Ma L. (2019). The TERT locus genotypes of rs2736100-CC/CA and rs2736098-AA predict shorter survival in renal cell carcinoma. Urol. Oncol..

[16] Lu Y., Yan C., Du J., Ji Y., Gao Y., Zhu X., Yu F., Huang T., Dai J., Ma H. (2017). Genetic variants affecting telomere length are associated with the prognosis of esophageal squamous cell carcinoma in a Chinese population. Mol. Carcinog..

[17] Chen Z., Wang J., Bai Y., Wang S., Yin X., Xiang J., Li X., He M., Zhang X., Wu T. (2017). The associations of TERT-CLPTM1L variants and TERT mRNA expression with the prognosis of early stage non-small cell lung cancer. Cancer Gene Ther..

[18] Jannuzzi A.T., Karaman E., Oztas E., Yanar H.T., Özhan G. (2015). Telomerase Reverse Transcriptase (TERT) Gene variations and Susceptibility of Colorectal Cancer. Genet. Test Mol. Bioma..

[19] Hofer P., Baierl A., Bernhart K., Leeb G., Mach K., Micksche M., Gsur A. (2012). Association of genetic variants of human telomerase with colorectal polyps and colorectal cancer risk. Mol. Carcinog..

[20] Kinnersley B., Migliorini G., Broderick P., Whiffin N., Dobbins S.E., Casey G., Hopper J., Sieber O., Lipton L., Kerr D.J. (2012). Colon Cancer Family Registry. The TERT variant rs2736100 is associated with colorectal cancer risk. Br. J. Cancer.

[21] Bailey C.E., Hu C.Y., You Y.N., Bednarski B.K., Rodriguez-Bigas M.A., Skibber J.M., Cantor S.B., Chang G.J. (2015). Increasing disparities in the age-related incidences of colon and rectal cancers in the United States, 1975–2010. JAMA Surg..

[22] Ryan J.E., Warrier S.K., Lynch A.C., Heriot A.G. (2015). Assessing pathological complete response to neoadjuvant chemoradiotherapy in locally advanced rectal cancer: A systematic review. Color. Dis..

[23] Fokas E., Liersch T., Fietkau R., Hohenberger W., Beissbarth T., Hess C., Becker H., Ghadimi M., Mrak K., Merkel S. (2014). Tumor regression grading after preoperative chemoradiotherapy for locally advanced rectal carcinoma revisited: Updated results of the CAO/ARO/AIO-94 trial. J. Clin. Oncol..

[24] Rampazzo E., Del Bianco P., Bertorelle R., Boso C., Perin A., Spiro G., Bergamo F., Belluco C., Buonadonna A., Palazzari E. (2018). The predictive and prognostic potential of plasma telomerase reverse transcriptase (TERT) RNA in rectal cancer patients. Br. J. Cancer.

[25] Pucciarelli S., Rampazzo E., Briarava M., Maretto I., Agostini M., Digito M., Keppel S., Friso M.L., Lonardi S., De Paoli A. (2012). Telomere-specific reverse transcriptase (hTERT) and cell-free RNA in plasma as predictors of pathologic tumor response in rectal cancer patients receiving neoadjuvant chemoradiotherapy. Ann. Surg. Oncol..

[26] Snetselaar R., van Oosterhout M.F.M., Grutters J.C., van Moorsel C.H.M. (2018). Telomerase Reverse Transcriptase Polymorphism rs2736100: A Balancing Act between Cancer and Non-Cancer Disease, a Meta-Analysis. Front. Med..

[27] Ge M., Shi M., An C., Yang W., Nie X., Zhang J., Lv Z., Li J., Zhou L., Du Z. (2016). Functional evaluation of TERT-CLPTM1L genetic variants associated with susceptibility of papillary thyroid carcinoma. Sci. Rep..

[28] Zou P., Gu A., Ji G., Zhao L., Zhao P., Lu A. (2012). The TERT rs2736100 polymorphism and cancer risk: A meta-analysis based on 25 case-control studies. BMC Cancer.

[29] Gallicchio L., Gadalla S.M., Murphy J.D., Simonds N.I. (2018). The Effect of Cancer Treatments on Telomere Length: A Systematic Review of the Literature. J. Natl. Cancer Inst..

[30] Diker-Cohen T., Uziel O., Szyper-Kravitz M., Shapira H., Natur A., Lahav M. (2013). The effect of chemotherapy on telomere dynamics: Clinical results and possible mechanisms. Leuk. Lymphoma.

[31] Garg M.B., Lincz L.F., Adler K., Scorgie F.E., Ackland S.P., Sakoff J.A. (2012). Predicting 5-fluorouracil toxicity in colorectal cancer patients from peripheral blood cell telomere length: A multivariate analysis. Br. J. Cancer.

[32] Longley D.B., Harkin D.P., Johnston P.G. (2003). 5-fluorouracil: Mechanisms of action and clinical strategies. Nat. Rev. Cancer.

[33] Engelhardt M., Ozkaynak M.F., Drullinsky P., Sandoval C., Tugal O., Jayabose S., Moore M.A. (1998). Telomerase activity and telomere length in pediatric patients with malignancies undergoing chemotherapy. Leukemia.

[34] Jia H., Wang Z. (2016). Telomere Length as a Prognostic Factor for Overall Survival in Colorectal Cancer Patients. Cell Physiol. Biochem..

[35] Barczak W., Sobecka A., Golusinski P., Masternak M.M., Rubis B., Suchorska W.M., Golusinski W. (2018). hTERT gene knockdown enhances response to radio- and chemotherapy in head and neck cancer cell lines through a DNA damage pathway modification. Sci. Rep..

[36] Giunco S., Zangrossi M., Dal Pozzolo F., Celeghin A., Ballin G., Petrara M.R., Amin A., Argenton F., Godinho Ferreira M., De Rossi A. (2020). Anti-Proliferative and Pro-Apoptotic Effects of Short-Term Inhibition of Telomerase In Vivo and in Human Malignant B Cells Xenografted in Zebrafish. Cancers.

[37] Ségal-Bendirdjian E., Geli V. (2019). Non-canonical Roles of Telomerase: Unraveling the Imbroglio. Front. Cell Dev. Biol..

[38] Martínez P., Blasco M.A. (2011). Telomeric and extra-telomeric roles for telomerase and the telomere-binding proteins. Nat. Rev. Cancer.

[39] Edge S.B., Compton C.C. (2010). The American Joint Committee on Cancer: The 7th Edition of the AJCC Cancer Staging Manual and the Future of TNM. Ann. Surg. Oncol..

[40] Amin M.B., Greene F.L., Edge S.B., Compton C.C., Gershenwald J.E., Brookland R.K., Meyer L., Gress D.M., Byrd D.R., Winchester D.P. (2017). The Eighth Edition AJCC Cancer Staging Manual: Continuing to build a bridge from a population-based to a more “personalized” approach to cancer staging. CA Cancer J. Clin..

[41] Mandard A.M., Dalibard F., Mandard J.C., Marnay J., Henry-Amar M., Petiot J.F., Roussel A., Jacob J.H., Segol P., Samama G. (1994). Pathologic assessment of tumor regression after preoperative chemoradiotherapy of esophageal carcinoma. Clinicopathologic correlations. Cancer.

[42] Boscolo-Rizzo P., Giunco S., Rampazzo E., Brutti M., Spinato G., Menegaldo A., Stellin M., Mantovani M., Bandolin L., Rossi M. (2020). TERT promoter hotspot mutations and their relationship with TERT levels and telomere erosion in patients with head and neck squamous cell carcinoma. J. Cancer Res. Clin. Oncol..

[43] Gianesin K., Noguera-Julian A., Zanchetta M., Del Bianco P., Petrara M.R., Freguja R., Rampon O., Fortuny C., Camós M., Mozzo E. (2016). Premature aging and immune senescence in HIV-infected children. AIDS.

[44] Rampazzo E., Bertorelle R., Serra L., Terrin L., Candiotto C., Pucciarelli S., Del Bianco P., Nitti D., De Rossi A. (2010). Relationship between telomere shortening, genetic instability, and site of tumour origin in colorectal cancers. Br. J. Cancer.

[45] Solé X., Guinó E., Valls J., Iniesta R., Moreno V. (2006). SNPStats: A web tool for the analysis of association studies. Bioinformatics.

[46] Grambsch P.M., Therneau T.M. (1994). Proportional hazards tests and diagnostics based on weighted residuals. Biometrika.

