# Supplementary material for: Genetic Variants of the TERT Gene, Telomere Length, and Circulating TERT as Prognostic Markers in Rectal Cancer Patients
Source: Cancers (Basel). 2020 Oct 25;12(11):3115. doi: 10.3390/cancers12113115 (PMC7692334; doi:10.3390/cancers12113115)
Supplement: Supplementary file 1 [file cancers-12-03115-s001.pdf]

cancers

Supplementary Materials: Genetic Variants of the *TERT* Gene, Telomere Length, and Circulating *TERT* as Prognostic Markers in Rectal Cancer Patients

Enrica Rampazzo, Erika Cecchin, Paola Del Bianco, Chiara Menin, Gaya Spolverato, Silvia Giunco, Sara Lonardi, Sandro Malacrida, Antonino De Paoli, Giuseppe Toffoli, Salvatore Pucciarelli and Anita De Rossi

Table S1A. Association between SNP genotypes and RTL in peripheral blood cells after neoadjuvant therapy.

SNP	Genotype	n	RTL median (IQR)	*p-value* *
**rs2736108**	TT	8	1.05 (0.95 to 1.14)	*Ref*
	TC	27	1.17 (1.00 to 1.36)	*0.167*
	CC	36	1.13 (1.03 to 1.28)	*0.238*
rs2735940	GG	15	1.09 (1.00 to 1.31)	*Ref*
	AG	31	1.15 (1.05 to 1.29)	*0.526*
	AA	13	1.06 (0.96 to 1.16)	*0.390*
rs2736098	TT	4	1.01 (0.95 to 1.08)	*Ref*
	TC	19	1.15 (1.00 to 1.26)	*0.258*
	CC	49	1.15 (1.03 to 1.28)	*0.166*
rs2736100	CC	19	1.06 (0.95 to 1.21)	*Ref*
	AC	34	1.15 (1.09 to 1.28)	*0.277*
	AA	17	1.11 (1.02 to 1.32)	*0.530*
rs35241335	TT	67	1.14 (0.99 to 1.26)	*Ref*
	TG	5	1.28 (1.27 to 1.30)	***0.039***
	GG	1	1.41	*0.134*
rs11742908	CC	40	1.16 (1.03 to 1.32)	*Ref*
	CG	23	1.15 (0.95 to 1.26)	*0.191*
	GG	2	1.11 (1.11 to 1.12)	*0.651*
rs2736122	GG	45	1.15 (1.05 to 1.30)	*Ref*
	AG	25	1.10 (0.96 to 1.21)	*0.240*
	AA	2	1.20 (1.05 to 1.36)	*0.733*
rs2853690	GG	50	1.14 (1.00 to 1.28)	*0.350*
	AG	18	1.09 (0.97 to 1.21)	*Ref*
	AA	2	1.20 (1.05 to 1.36)	*0.430*

RTL = relative telomere length; IQR= interquartile range; * adjusted by age.

Table S1B. Association between SNP genotypes and RTL changes after neoadjuvant therapy.

SNP	Genotype	n	ΔRTL (T2-T0) median (IQR)	*p-value* *
**rs2736108**	TT	8	-0.35 (-0.65 to 0.20)	*Ref*
	TC	27	-0.04 (-0.29 to 0.16)	*0.171*
	CC	36	0.17 (-0.11 to 0.29)	***0.020***
rs2735940	GG	15	0.17 (-0.05 to 0.32)	*Ref*
	AG	31	0.02 (-0.12 to 0.33)	*0.535*
	AA	13	-0.33 (-0.68 to 0.26)	***0.008***
rs2736098	TT	4	-0.18 (-0.65 to 0.20)	*Ref*
	TC	19	-0.09 (-0.33 to 0.29)	*0.395*
	CC	49	0.10 (-0.13 to 0.26)	*0.215*
rs2736100	CC	19	-0.15 (-0.49 to 0.10)	*Ref*
	AC	34	0.16 (-0.13 to 0.36)	***0.006***

	AA	17	0.17 (0.00 to 0.24)	***0.007***
rs35241335	TT	67	0.00 (-0.22 to 0.26)	*Ref*
	TG	5	0.26 (0.15 to 0.33)	*0.379*
	GG	1	0.35	*0.381*
rs11742908	CC	40	0.13 (-0.14 to 0.26)	*Ref*
	CG	23	-0.10 (-0.35 to 0.27)	*0.276*
	GG	2	0.12 (0.003 to 0.25)	*0.803*
rs2736122	GG	45	0.01 (-0.32 to 0.26)	*Ref*
	AG	25	0.1 (-0.15 to 0.26)	*0.793*
	AA	2	-0.04 (-0.07 to -0.02)	*0.922*
rs2853690	GG	50	0.01 (-0.33 to 0.26)	*0.552*
	AG	18	0.11 (-0.13 to 0.28)	*Ref*
	AA	2	-0.04 (-0.07 to -0.02)	*0.852*

RTL = relative telomere length; IQR= interquartile range; * adjusted by age.

Table S2A. Association between SNP genotypes and *TERT* levels after neoadjuvant therapy.

SNP	Genotype	n	*TERT* median (IQR)	*p-value*
**rs2736108**	TT	12	135.5 (82.5 to 226.2)	*Ref*
	TC	42	37.5 (0.0 to 142.2)	*0.069*
	CC	61	40.0 (0.0 to 177.0)	*0.088*
rs2735940	GG	28	85.0 (0.0 to 184.0)	*Ref*
	AG	49	30.0 (0.0 to 167.0)	*0.468*
	AA	26	49.5 (0.0 to 183.0)	*0.889*
rs2736098	TT	9	122.0 (0.0 to 226.0)	*Ref*
	TC	31	53.0 (0.0 to 143.0)	*0.360*
	CC	77	35.0 (0.0 to 177.0)	*0.339*
rs2736100	CC	30	49.5 (0.0 to 174.5)	*Ref*
	AC	56	32.5 (0.0 to 152.7)	*0.82*
	AA	26	85.0 (0.0 to 188.0)	*0.643*
rs35241335	TT	108	53.5 (0.0 to 177.7)	*Ref*
	TG	10	0.0 (0.0 to 35.5)	*0.230*
	GG	1	90.0	*0.986*
rs11742908	CC	57	40.0 (0.0 to 167.0)	*Ref*
	CG	53	55.0 (0.0 to 177.0)	*0.659*
	GG	2	0.0 (0.0 to 0.0)	*0.287*
rs2736122	GG	71	42.0 (0.0 to 154.0)	*Ref*
	AG	43	45.0 (0.0 to 218.5)	*0.437*
	AA	5	0.0 (0.0 to 144.0)	*0.672*
rs2853690	GG	80	25.0 (0.0 to 134.2)	***0.016***
	AG	30	131.0 (0.0 to 229.5)	*Ref*
	AA	4	0.0 (0.0 to 36.0)	*0.108*

IQR= interquartile range

Table S2B. Association between SNP genotypes and *TERT* changes after neoadjuvant therapy.

SNP	Genotype	n	**Δ*TERT* (T2-T0) median (IQR)**	*p-value*
**rs2736108**	TT	12	7.5 (-223.5 to 138.7)	*Ref*
	TC	42	0.0 (-178.5 to 59.2)	*0.856*
	CC	60	0.0 (-117.7 to 27.0)	*0.844*
rs2735940	GG	28	0.0 (-96.2 to 72.5)	*Ref*
	AG	48	0.0 (-157.7 to 20.0)	*0.441*
	AA	26	-97.5 (-206.2 to 134.7)	*0.425*
rs2736098	TT	9	-87.0 (-183.0 to 133.0)	*Ref*
	TC	31	0.0 (-176.0 to 113.0)	*0.932*
	CC	76	0.0 (-117.7 to 20.5)	*0.943*
rs2736100	CC	30	-38.0 (-198.0 to 9.0)	*Ref*
	AC	55	0.0 (-133.0 to 78.0)	*0.414*
	AA	26	0.0 (-123.2 to 33.7)	*0.751*

rs35241335	TT	107	0.0 (-128.5 to 56.0)	*Ref*
	TG	10	-105.0 (-211.7 to -15.5)	*0.092*
	GG	1	-95.0	*0.676*
rs11742908	CC	56	0.0 (-207.5 to 23.0)	*Ref*
	CG	53	0.0 (-126.0 to 101.0)	*0.162*
	GG	2	0.0 (0.0 to 0.0)	*0.602*
rs2736122	GG	70	0.0 (-103.7 to 90.2)	*Ref*
	AG	43	-5.0 (-225.0 to 11.0)	*0.126*
	AA	5	0.0 (-415.0 to 95.0)	*0.683*
rs2853690	GG	79	0.0 (-160.5 to 54.0)	*0.463*
	AG	30	0.0 (-57.5 to 33.7)	*Ref*
	AA	4	-207.5 (-435.0 to 23.7)	*0.066*

IQR= interquartile range

Table S3A. Univariate logistic regression analysis of clinical and demographical characteristics according to TRG probability in rectal cancer patients.

**Characteristics**		**TRG 1-2 (%) (n = 90)**	**TRG 3-5 (%) (n = 104)**	**OR TRG 1-2 vs TRG 3-5 (95% CI)**	***p-value***
Age (years)	cont.			1.03 (1—1.06)	***0.043***
Gender	M	65 (72.2)	73 (70.2)	1.1 (0.6—2.1)	*0.756*
	F	25 (27.8)	31 (29.8)	*Ref*	
Distance from anal verge(cm)	Median (IQR)	5 (3—8)	5 (4—8)	0.95 (0.85—1.06)	*0.369*
CEA, ng/ml (T0)	Median (IQR)	2.0 (1.0—3.1)	2.2 (1.2—4.0)	1.0 (0.96—1.03)	*0.828*
Grading	G1	3 (4.5)	9 (10.1)	*Ref*	
	G2	59 (88.1)	73 (82.0)	2.4 (0.6—9.4)	*0.199*
	G3	5 (7.5)	7 (7.9)	2.1 (0.4—12.2)	*0.390*
cTNM	I	5 (5.7)	1 (1.0)	*Ref*	
	II	11 (12.5)	10 (9.9)	0.2 (0.0—2.2)	*0.199*
	III	70 (79.5)	89 (88.1)	0.2 (0.0—1.4)	*0.095*
	IV	2 (2.3)	1 (1.0)	0.4 (0.0—10.0)	*0.577*
Total RT dose	<50.4 Gy	10 (12.5)	12 (12.2)	*Ref*	
	50.4 Gy	42 (52.5)	47 (48.0)	1.1 (0.4—2.7)	*0.884*
	>50.4 Gy	28 (35.0)	39 (39.8)	0.9 (0.3—2.3)	*0.763*
Fluoropyrimide	Alone	44 (53.7)	49 (48.5)	*Ref*	
	+ other drugs	36 (43.9)	48 (47.5)	0.8 (0.5—1.5)	*0.552*
	No	2 (2.4)	4 (4.0)	0.6 (0.1—3.2)	*0.511*

OR = odds ratio; TRG = tumor regression grade; CI = confidence interval; cont. = continuous variable; CEA = carcinoembryonic antigen; cTNM = clinical Tumor, Node, Metastasis classification; RT= radiotherapy.

Table S3B. Univariate logistic regression analysis of covariates of interest according to TRG probability in rectal cancer patients.

		**TRG 1–2 (%)**	**TRG 3–5 (%)**	**OR TRG 1–2 vs TRG 3–5 (95% CI)**	***p-value***
rs2736108	TT	3 (3.4)	13 (13.3)	*Ref*	
	TC	29 (33.0)	35 (35.7)	3.6 (0.9—13.8)	*0.063*
	CC	56 (63.6)	50 (51.0)	4.8 (1.3—18.0)	***0.018***
rs2735940	AA	13 (16.5)	25 (28.1)	*Ref*	
	AG	40 (50.6)	44 (49.4)	1.7 (0.8—3.9)	*0.169*
	GG	26 (32.9)	20 (22.5)	2.5 (1.0—6.1)	***0.043***
rs2736098	TT	2 (2.2)	10 (10.1)	*Ref*	
	TC	22 (24.7)	29 (29.3)	3.8 (0.7—19.1)	*0.106*
	CC	65 (73.0)	60 (60.6)	5.4 (1.1—25.7)	***0.034***
rs2736100	CC	18 (21.4)	26 (26.5)	*Ref*	*Ref*
	AC	44 (52.4)	51 (52.0)	1.2 (0.6—2.6)	*0.551*

	AA	22 (26.2)	21 (21.4)	1.5 (0.7—3.5)	*0.338*
rs35241335	TT	80 (88.9)	89 (89.0)	*Ref*	
	TG	9 (10.0)	11 (11.0)	0.9 (0.4—2.3)	*0.843*
	GG	1 (1.1)	0 (0)		
rs11742908	CC	49 (57.0)	54 (57.4)	*Ref*	
	CG	35 (40.7)	38 (40.4)	1.0 (0.6—1.9)	*0.961*
	GG	2 (2.3)	2 (2.1)	1.1 (0.2—8.1)	*0.924*
rs2736122	GG	49 (58.3)	61 (60.4)	Ref	
	AG	29 (34.5)	36 (35.6)	1.0 (0.5—1.9)	*0.993*
	AA	6 (7.1)	4 (4.0)	1.9 (0.5—7.0)	*0.354*
rs2853690	GG	64 (73.6)	65 (67.0)	1.9 (0.9-3.7)	*0.085*
	AG	16 (18.4)	30 (30.9)	*Ref*	
	AA	7 (8.0)	2 (2.1)	6.6 (1.2—35.4)	***0.029***
RTL (T0)	≤median (1.055)	45 (50.0)	52 (50.0)	*Ref*	
	>median	45 (50.0)	52 (50.0)	1.0 (0.6—1.8)	*1.000*
RTL (T2)	≤median (1.14)	14 (43.8)	24 (57.1)	*Ref*	
	>median	18 (56.2)	18 (42.9)	1.7 (0.7—4.3)	*0.255*
ΔRTL (T2-T0)	≤0 (stable/decrease)	17 (53.1)	18 (42.9)	*Ref*	
	>0 (increase)	15 (46.9)	24 (57.1)	0.7 (0.3—1.7)	*0.382*
TERT (T0)	≤median (122)	29 (50.0)	43 (50.6)	*Ref*	
	>median	29 (50.0)	42 (49.4)	1.0 (0.5—3.0)	*0.945*
TERT (T2)	≤median (45)	40 (76.9)	22 (31.0)	7.4 (3.3—16.8)	***<0.001***
	>median	12 (23.1)	49 (69.0)	*Ref*	
Δ*TERT* (T2-T0)	≤0 (stable/decrease)	46 (90.2)	33 (46.5)	10.6 (3.8—29.8)	***<0.001***
	>0 (increase)	5 (9.8)	38 (53.5)	*Ref*	

RTL = relative telomere length; TRG = tumor regression grade; OR = odds ratio; CI = confidence interval.

Table S4A. Haplotype distribution in responder (TRG 1-2) and non-responders (TRG 3-5) to CRT.

SNP Haplotype id	rs2736108	rs2735940	rs2736098	rs2736100	rs35241335	rs11742908	rs2736122	rs2853690	TRG 1-2 % *	TRG 3-5 % *	OR* TRG 1-2 vs TRG 3-5 (95% CI)	*p-value†*
1	C	G	C	A	T	C	G	G	0.22	0.15	1	---
2	C	G	C	A	T	C	A	A	0.11	0.13	0.93 (0.39—2.22)	0.87
3	T	A	T	C	T	C	G	G	0.07	0.14	0.45 (0.14—1.49)	0.19
4	C	A	C	C	T	C	G	G	0.09	0.11	0.90 (0.31—2.61)	0.85
5	C	A	C	C	T	G	G	G	0.05	0.07	0.65 (0.21—2.04)	0.47
6	T	A	C	C	T	C	G	G	0.06	0.07	1.05 (0.33—3.31)	0.93
7	C	G	C	A	T	G	G	G	0.05	0.06	1.08 (0.27—4.40)	0.91
8	C	G	C	A	G	C	G	G	0.05	0.04	1.41 (0.44—4.48)	0.56
9	C	G	C	C	T	C	G	G	0.03	0.04	1.13 (0.20—6.48)	0.89
**10**	**C**	**G**	**C**	**A**	**T**	**C**	**A**	**G**	**0.04**	**0.01**	**6.80 (1.04—44.41)**	**0.05**
11	T	A	T	C	T	G	G	G	0.06	0.01	1.26 (0.18—8.62)	0.82
12	C	G	C	C	T	G	G	G	0.03	0.02	10.18 (0.00—20844.24)	0.55
rare	*	*	*	*	*	*	*	*	NA	NA	0.83 (0.36—1.87)	0.65

* frequencies are estimated by SNPStats program; TRG = tumor regression grade; OR = odd ratio; CI= confidence interval; † adjusted by age

Table S4B. 5' *TERT* gene haplotype distribution in responders (TRG 1-2) and non-responders (TRG 3-5) to CRT.

SNP Haplotype id	rs2736108	rs2735940	rs2736098	TRG 1-2* %	TRG 3-5* %	OR* TRG 1-2 vs TRG 3-5 (95% CI)	*p-value†*
1	C	G	C	0.59	0.46	1	---
2	C	A	C	0.19	0.21	0.69 (0.38—1.27)	0.24
**3**	**T**	**A**	**T**	**0.13**	**0.24**	**0.45 (0.26—0.80)**	**0.007**
4	T	A	C	0.07	0.08	0.74 (0.33—1.64)	0.46
rare	*	*	*	NA	NA	1.98 (0.20—19.52)	0.56

* frequencies are estimated by SNPStats program; TRG= tumor regression grade; OR= odd ratio; CI= confidence interval; † adjusted by age.

Table S5. Univariate Cox regression analysis of EFS.

		E/N	HR (95%CI)	*p-value*
Age at diagnosis (years)	cont.		1.00 (0.98—1.03)	*0.934*
Gender	M	45/136	1.0 (0.6—1.7)	*0.955*
	F	19/56	*Ref*	
rs2736108	CC	36/104	*Ref*	
	TC	20/64	0.9 (0.5—1.5)	*0.668*
	TT	6/16	1.1 (0.5—2.6)	*0.806*
rs2735940	GG	13/44	*Ref*	
	AG	33/84	1.3 (0.7—2.4)	*0.444*
	AA	11/38	0.9 (0.4—2.1)	*0.901*
rs2736098	CC	43/123	*Ref*	
	TC	15/51	0.8 (0.5—1.5)	*0.524*
	TT	3/12	0.7 (0.2—2.4)	*0.619*
rs2736100	CC	18/44	*Ref*	
	AC	31/94	0.7 (0.4—1.3)	*0.291*
	AA	12/42	0.6 (0.3—1.3)	*0.236*
rs35241335	TT	59/168	*Ref*	
	TG	3/19	0.4 (0.1—1.3)	*0.128*
	GG	0/1		
rs11742908	CC	34/103	*Ref*	
	CG	22/71	0.9 (0.5—1.5)	*0.679*
	GG	2/4	1.5 (0.4—6.4)	*0.554*
rs2736122	GG	36/108	*Ref*	
	AG	22/65	1.0 (0.6—1.6)	*0.878*
	AA	3/10	0.7 (0.2—2.4)	*0.606*
rs2853690	GG	36/127	0.7 (0.4—1.1)	*0.134*
	AG	19/46	*Ref*	
	AA	3/9	0.7 (0.2—2.5)	*0.631*
RTL (T0)	≤median (1.055)	29/95	*Ref*	
	>median	35/97	1.1 (0.7—1.8)	*0.670*
RTL (T2)	≤median (1.14)	17/38	*Ref*	
	>median	7/36	0.4 (0.1—0.9)	***0.026***
ΔRTL (T2-T0)	≤0 (stable/decrease)	16/35	*Ref*	
	>0 (increase)	8/39	0.4 (0.2—1.0)	***0.049***
TERT (T0)	≤median (122)	18/70	*Ref*	
	>median	26/71	1.5 (0.8—2.8)	*0.171*
TERT (T2)	≤median (45)	12/62	0.4 (0.2—0.8)	***0.012***
	>median	25/60	*Ref*	
Δ*TERT* (T2-T0)	≤0 (stable/decrease)	19/79	0.4 (0.2—0.9)	***0.016***
	>0 (increase)	18/42	*Ref*	

EFS = Event –free Survival; HR = hazard ratio; cont. = continuous variable; CI = Confidence Interval; RTL = relative telomere length.

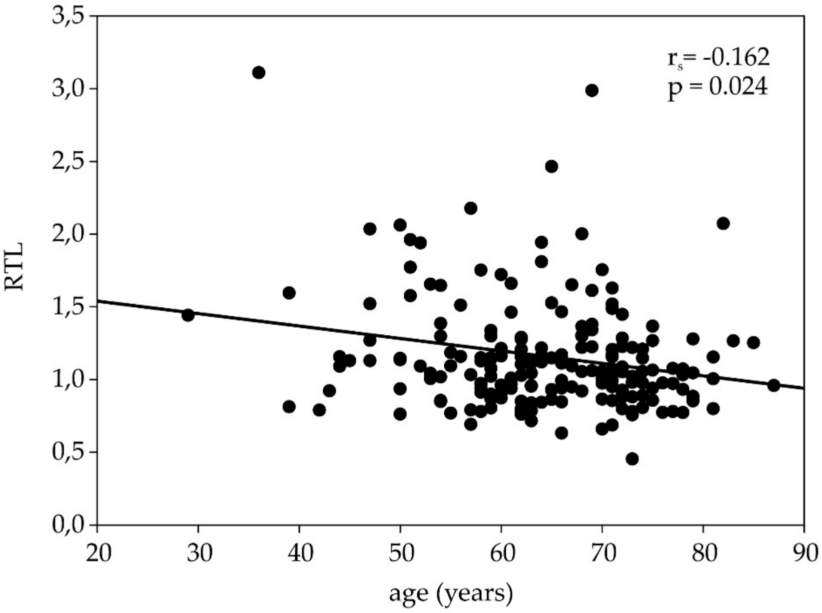

Figure S1. Correlation between age and telomere length in peripheral blood cells of rectal cancer patients.

© 2020 by the authors. Licensee MDPI, Basel, Switzerland. This article is an open access article distributed under the terms and conditions of the Creative Commons Attribution (CC BY) license (http://creativecommons.org/licenses/by/4.0/).
